# Delayed presentation to hospital care is associated with sequelae but not mortality in children with cerebral malaria in Malawi

**DOI:** 10.1186/s12936-022-04080-2

**Published:** 2022-02-22

**Authors:** Arabella Borgstein, Bo Zhang, Colin Lam, Montfort Bernard Gushu, Alice Wangui Liomba, Albert Malenga, Paul Pensulo, Andrew Tebulo, Dylan S. Small, Terrie Taylor, Karl Seydel

**Affiliations:** 1 Blantyre Malaria Project, Kamuzu University of Health Sciences, Private Bag 360, Blantyre, Malawi; 2 Department of Statistics and Data Science, The Wharton School, University of Pennsylvania, Philadelphia, USA; 3 Bronx High School of Science, Bronx, NY USA; 4 Department of Osteopathic Medical Specialties, College of Osteopathic Medicine, Michigan State University, East Lansing, MI USA; 5 Malawi-Liverpool-Wellcome Trust Research Programme, Kamuzu University of Health Sciences, Blantyre, Malawi; 6 Present Address: St. George’s University of London/University of Nicosia Medical School, Nicosia, Cyprus

**Keywords:** Cerebral malaria, Neurological sequelae, Delay to presentation

## Abstract

**Background:**

Cerebral malaria is still a major cause of death in children in sub-Saharan Africa. Among survivors, debilitating neurological sequelae can leave children with permanent cognitive impairments and societal stigma, resulting in taxing repercussions for their families. This study investigated the effect of delay in presentation to medical care on outcome in children with cerebral malaria in Malawi.

**Methods:**

This retrospective study included participants enrolled in a longstanding study of cerebral malaria between 2001 and 2021 and considered coma duration prior to arrival at hospital (with or without anti-malarial treatment), HIV status, blood lactate levels at admission and age as factors that could affect clinical outcome. Outcomes were categorized as full recovery, sequelae at the time of discharge, or death. A multinomial regression was fit and run controlling for coma duration, HIV status, lactate levels and age, to determine the association between each explanatory variable and outcome.

**Results:**

A total of 1663 children with cerebral malaria, aged 6 months to 14 years were included. Longer coma duration (in hours) was associated with greater odds of developing sequelae (OR = 1.023, 95% CI 1.007–1.039, p = 0.006) but not death (OR = 1.00, 95% CI 0.986–1.015, p = 0.961). Younger age (in months) was also correlated with higher rates of sequelae, (OR = 0.990, 95% CI 0.983–0.997, p = 0.004) but not with increased mortality (OR = 0.998, 95% CI 0.993–1.003, p = 0.335). Blood lactate levels on admission were correlated with mortality (OR = 1.125, 95% CI 1.090–1.161, p < 0.001) but not associated with increased rates of sequelae (OR = 1.016, 95% CI 0.973–1.060, p = 0.475). Positive HIV status and treatment with an anti-malarial (artemisinin or non-artemisinin-based) prior to arrival at the hospital were not significantly associated with either adverse outcome.

**Conclusions:**

In Malawian children with cerebral malaria, higher rates of sequelae were significantly associated with extended coma duration prior to admission and younger age. Mortality rates were correlated with increased lactate levels on admission. The differential effects of variables on clinical outcomes suggest that there may be different pathogenic pathways leading to sequelae and death. Actions taken by parents and health care professionals are critical in defining when patients arrive at hospital and determining their ultimate outcome.

**Supplementary Information:**

The online version contains supplementary material available at 10.1186/s12936-022-04080-2.

## Background

Cerebral malaria (CM) is the gravest manifestation of severe malaria and results in the highest mortality rates encountered in this disease [1, 2]. It is defined by peripheral asexual *Plasmodium falciparum* parasitaemia and coma (Blantyre coma score ≤ 2) persisting for more than 2 h after a seizure and with no other identifiable cause [3]. In malaria-endemic areas children are more vulnerable than adults, with 5% of all paediatric deaths being attributed to CM, making it the third major cause of death in children under the age of 5 worldwide [4]. Even with timely and appropriate care, mortality due to CM remains between 10 and 20% [3]. In survivors, there can be serious neurological sequelae that leave children with permanent cognitive impairments [5].

Neurological sequelae have been reported in 9–23% of paediatric CM survivors at discharge [6–8]: 21–23% have been found to develop cognitive deficits or epilepsy in the months after discharge [9, 10] and neurodevelopmental impairments have been reported to accumulate for up to one year after hospital discharge. As a result, more than 50% of CM survivors eventually develop some form of deficit [11]. These neurosequelae can manifest in a variety of ways including compromised hearing, sight or speech, motor abnormalities, paralysis, learning and memory defects, disruptive behaviours, and epilepsy [12–14]. In sub-Saharan Africa, the appropriate resources for support, treatment, and rehabilitation of children with these sequelae are scarce. In addition, these disabilities still carry heavy societal stigma and can have devastating repercussions on children and their families within their communities.

Although there has been substantial research on the factors associated with malaria mortality, factors associated with neurosequelae have been less well documented [11]. A study in The Gambia showed that post-CM deaths were associated with hypoglycaemia and acidosis, whereas neurological sequelae were associated with repeated seizures and deep prolonged coma [15]. This suggests that neurological disability could be associated with prolonged duration of symptoms and that the causal pathways of mortality and sequelae may be different. No research has considered the delay of presentation to medical care—with its associated extended duration of symptoms—and its direct impact on clinical outcome in CM. Actions taken before a child reaches the hospital, such as the rapidity of care-seeking by the parents or guardians and the availability of treatment options at the local level, may be critical to disease outcome. Death and sequelae may have different pathogenic pathways and this concept has been little explored.

To address these questions, data from a longstanding study of CM were used to investigate the contribution of coma duration prior to admission (a proxy measure for delay to presentation at hospital and receipt of treatment) to the distinct clinical outcomes of full recovery, sequelae, or death. The data spanned 20 years of research in a central government hospital of Malawi. The exposure variables were chosen based on a previous Malawi-based study investigating contributors to mortality which found lactate to be the only independent predictor of death [16]. In this previous study coma duration was included in the variables of interest; however, the outcomes were binary only (survival or death) without consideration of survival with sequelae. The study found that HIV status was not related to mortality although it has been shown to be a known risk factor for complicated malaria, positive status being associated with an increase in incidence and severity and decrease in treatment success [17–20]. Other previous studies have revealed that age and duration of coma symptoms are risk factors for cognitive impairment post-CM [9–11, 21, 22]. In addition to these exposure variables, pre-treatment with artesunate or non-artesunate based therapies was included given the recent emergence of readily available artemisinin-based combination therapy (ACT) in the community.

## Methods

Data analysed were previously collected by the Blantyre Malaria Project (BMP) in the Paediatric Research Ward (PRW) of Queen Elizabeth Central Hospital (QECH), the largest government-run tertiary care hospital in Malawi. Only patients meeting the following definition of CM were included; Blantyre coma score ≤ 2 with no other identifiable cause of coma and *P. falciparum* parasitaemia on a peripheral blood smear.

Patients were given the maximum level of high-dependency care available and treated with antimalarial medication, antipyretics, antibiotics, and anticonvulsants according to the national guidelines at the time. Ethical approval for the study was provided by the institutional review boards at the University of Malawi College of Medicine in Blantyre, Malawi, and Michigan State University.

On admission, written informed consent was obtained from the parent or guardian of each child to authorize the collection and analysis of samples as well as access to deidentified data. Two rapid tests, Uni-Gold Recombigen HIV-1/2 (Trinity Biotech) and Determine HIV-1/2 (Inverness Medical), were used to diagnose infection with HIV, with a tiebreaker used if discrepant (Capillus, Trinity Biotech). Blood lactate levels were measured on admission using a Lactate Pro 2 point of care detector (Arkray Inc). A lumbar puncture was performed to rule out meningitis.

History of the disease episode was determined using a questionnaire completed by the nurse in consultation with the primary caregiver at the time of admission. This included details of symptom onset, when and where medical attention was sought and whether any medication was given prior to arrival at the hospital. Children were screened on admission for any pre-existing neurological condition; if present they were excluded from the study.

Clinical outcomes were determined by research clinicians at the time of discharge or death. Details of neurological sequelae were selected from a list (cognitive impairment, paresis, ataxia, aphasia, blindness, deafness, or behavioural changes), or, if not included in the list, described.

The statistical analysis included all subjects with a recorded outcome at discharge (full recovery, sequelae, or death). Missing data for other variables was imputed using multiple imputation with chained equations and five imputed datasets were created [23]. A proportional odds assumption was used to test the equality of the association between explanatory variables and outcome (full recovery, sequelae, and death) and assessed using a Brant test [24]. A multinomial regression was then fit to quantify the association between each variable and outcome. All analyses were performed on each imputed dataset and results were then pooled using standard methods [25]. Statistics were performed using the statistical computing software, R [26].

To understand the factors contributing to delayed presentation to medical care, the clinical notes of patients with coma duration of 24 h or longer (15% of the sample) were examined between 2002 and 2019.

## Results

Between 2001 and 2021, a total of 1663 children with cerebral malaria between the ages of 6 months and 14 years were admitted to the PRW (Table 1).

**Table 1:** Summary statistics for patients evaluated in study

Outcome	Overall (n = 1663)^a^	Full recovery (n = 1240)^a^	Sequelae (n = 146)^a^	Died (n = 277)^a^
Patient characteristics				
Coma duration prior to admission (hours)	7 (4, 16)	7 (4, 17)	8 (5, 21)	7 (4, 12)
Missing data	282	219	17	46
Age(months)	47.6 ± 29.5	48.7 ± 30.2	41.9 ± 23.8	45.5 ± 28.9
HIV (positive/total)	185/1440 (13%)	127/1062 (12%)	15/125 (12%)	43/253 (17%)
Missing data	223	178	21	24
Prior treatment				
Artemisinin-based therapy	396/1660 (24%)	304/1237 (25%)	31/146 (21%)	61/277 (22%)
Other	586/1660 (35%)	439/1237 (35%)	53/146 (36%)	94/277 (34%)
None	678/1660 (41%)	494/1237 (40%)	62/146 (42%)	122/277 (44%)
Missing data	3	3	0	0
Lactate concentration on admission (mmol/L)	5 (3, 10)	5 (3, 9)	5 (3, 9)	9 (5, 13)
Missing data	193	134	20	39

^a^Mean and standard deviation for age. Median and IQR for coma duration and lactate concentration. Numbers and percentages for HIV and prior treatment

### Associations with outcomes

A proportional odds assumption tested the hypothesis that the five variables evaluated had comparable effects on the three clinical outcomes. The proportional odds assumption was rejected at p = 0.05 level using an omnibus Brant test with a Chi-square statistic of 22.15 (6 degrees of freedom, p-value = 0.001) (Additional file 1: Table S1).

**Fig. 1:**
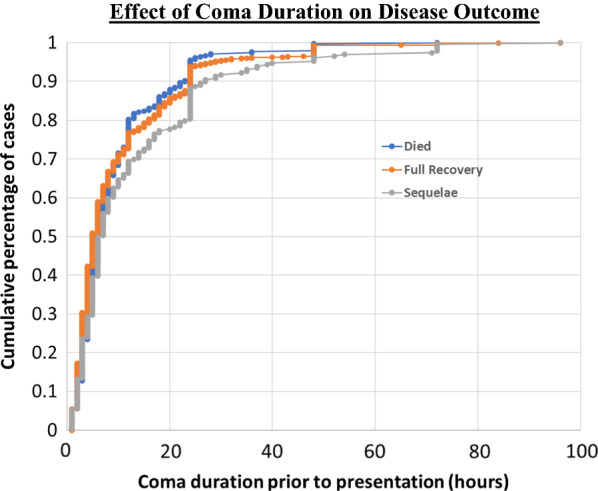
A cumulative distribution showing the effect of coma duration prior to presentation on the three clinical outcomes. As is also shown in the statistical analysis, longer coma duration is associated with higher rates of sequelae, compared to full recovery or death

Given the rejection of the proportional odds hypothesis, the contribution of each of the variables was then explored using a multinomial regression (Table 2). Longer coma duration prior to admission (OR 1.023, p-value = 0.006) was strongly associated with neurological sequelae, but not with death (OR 1.00, p-value = 0.961). This trend is highlighted in Fig. 1. Although the odds ratios appear minimal, this is partially due to the choice of units of hours. Were the consequences of a six-hour delay to have been considered rather than the delay of a single hour, the OR would increase to 1.14. Age was significantly and inversely associated with sequelae (OR 0.990, p = 0.004) but was not associated with death (OR 0.998, p = 0.335). Higher lactate levels were strongly correlated with death (OR 1.125, p < 0.001), but were not associated with sequelae (OR 1.016, p = 0.475). Being HIV positive and receiving prior treatment were not significantly associated with any outcome.

**Table 2:** Results of the multinomial regression showing the contribution of each exposure to outcome

Variable	Sequelae vs. full recovery	Death vs. full recovery
Age (months)	OR 0.990 [95% CI 0.983–0.997]; p = 0.004	OR 0.998 [95% CI 0.993–1.003]; p = 0.335
Coma duration (hours)	OR 1.023 [95% CI 1.007–1.039]; p = 0.006	OR 1.000 [95% CI 0.986–1.015]; p = 0.961
HIV status	OR 1.162 [95% CI 0.645–2.095]; p = 0.617	OR 1.324 [95% CI 0.880–1.991]; p = 0.179
Prior treatment: other	OR 0.968 [95% CI 0.652–1.436]; p = 0.870	OR 0.965 [95% CI 0.708–1.316]; p = 0.821
Prior treatment: artesunate	OR 0.788 [95% CI 0.486–1.278]; p = 0.333	OR 1.030 [95% CI 0.713–1.488]; p = 0.876
Lactate at admission (mmol/L)	OR 1.016 [95% CI 0.973, 1.060]; p = 0.475	OR 1.125 [95% CI 1.090–1.161]; p < 0.001

### Delay to presentation

**Fig. 2:**
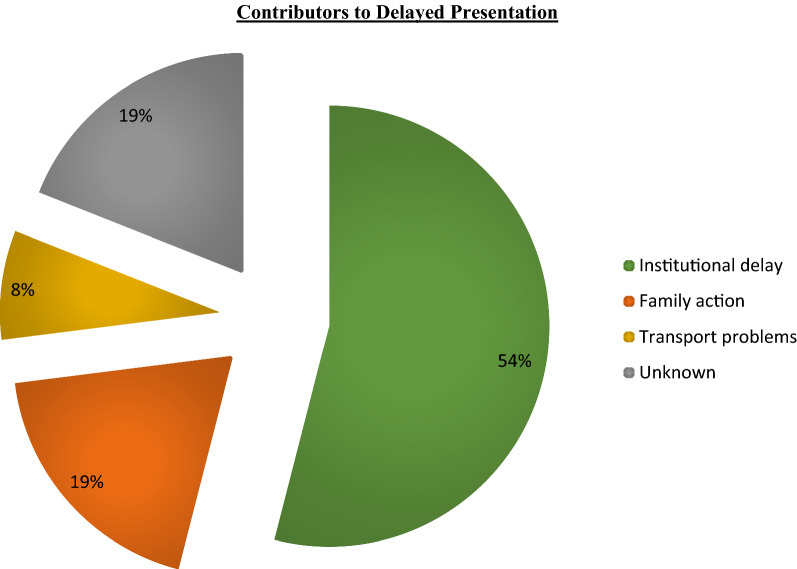
Proportions of primary reasons for delay to presentation in patients with coma duration ≥ 24 h prior to admission

Detailed patient histories were available from 2002 to 2019. Clinical notes of patients presenting with coma duration of 24 h or longer, and with sequelae at discharge were examined. Of these, fifty-seven had histories which included comments on delay to presentation. Delays were categorized in 1 of the following four categories (Table 3): institutional delay, family action, transport problems or unknown. Most of the analysed cases (54%) gave their reason for delay as being rooted in an institutional cause (Fig. 2, Table 3). Another 19% attributed their delay to the actions taken by the child’s parent or guardian and 8% to issues with transportation. For 19% no reasons could be identified.

**Table 3:** Definitions used to categorise delayed presentations

Institutional delay	The time taken for patients to reach the PRW from nationwide referral routes; local health centres, private clinics, district hospitals, Accident and Emergency, the Paediatric Special Care Ward, and other departments of QECH. This included the actions of health workers involved in the treatment and handling of patients prior to their arrival at the PRW, as well as protocol time and ambulance availability
Family action	The decisions made by the patients’ parent or guardian. These were often made based on the hope that the child would improve on their own or due to misinterpretation of the severity of their condition, sometimes believing they were “just sleeping”
Transport problems	In cases where patients lived in remote, rural areas, the lack of transport, lack of money for transport or sheer distance to travel to find transport were notable impeding factors
Unknown	The clinical notes did not detail (or the parent or guardian did not tell) the exact reason for delay in arrival at the PRW

## Discussion

Most families and communities in rural sub-Saharan Africa have little or no access to educational or rehabilitation resources and are ill-equipped to care for disabled children. If a child survives CM with severe, or even mild neurological deficits, their entire family may be subject to stigma, discrimination, and social isolation from their community [27]. In countries such as Malawi—where most of the population live below the poverty line—financial strain, cultural pressure and the burdens of daily life leave little capacity to cope with additional adversity. As well as alienating the family from their community, stigma and discrimination have been shown to have a detrimental effect on post-CM care of the patient [12].

There is an increasing awareness of the burden of neurological sequelae post-CM [28–30] however there remains a knowledge gap as to its pathogeneses, causes and clinical associations [2]. Previous studies have revealed that age, seizure frequency, prolonged fever and duration of coma symptoms are risk factors for cognitive impairment post-CM [9–11, 21, 22]. In this study coma duration prior to admission and younger age were identified as two significant drivers of neurosequelae in CM survivors (Fig. 2).

CM is typically more common in younger children. Other studies have also shown younger age to be associated with worse outcomes in CM survivors, children under the age of 5 showing considerably greater developmental delays one month after discharge, as well as more abnormal MRIs and associated impairment in cognitive ability, attention, and associative memory [21, 22]. Younger brains may be more vulnerable to CM as critical events in brain growth and development occur between birth and age 5 years [28]. It is uncertain, however, whether the increased neuroplasticity at this age facilitates more rapid recovery from injury, contributes to more serious and sustained neurological damage, or both [31–33].

As the disease is frequently lethal, patients with CM should be given the highest level of available attention, preferably in an intensive care unit or local equivalent [3, 14]. Immediate anti-malarial medication, antipyretics, and anticonvulsants should be administered, with ACT being regarded as the “first-line of defence” in the outpatient setting [5]. Clinical trials have established artemisinin-based combinations as being superior to other anti-malarials [34, 35] and they are now widely recommended as first-line drugs for treatment of malaria [3]. Although prior treatment with ACT did not affect clinical outcome in this study, it showed a trend toward decreasing sequelae, which was not seen with mortality. The lack of a statistically significant result could be due to the relatively recent introduction of outpatient ACT, which limited the number of cases in the analysis.

Lactic acidosis is frequently observed in patients with severe malaria and is a prognostic factor for both mortality and poor outcome, [6, 36–39] with prolonged periods of hyperlactataemia possibly leading to neuronal dysfunction as well as death [2]. A number of factors could potentially contribute to lactic acidosis in this setting, including parasite metabolism, aerobic glycolysis by activated immune cells, anaerobic glycolysis in hypoxic cells and tissues due to parasite sequestration and anaemia, and impeded clearance of lactate in the liver or kidneys [39]. Studies in Ghana and Thailand showed that dichloroacetate decreased lactate levels but did not specifically decrease overall mortality in severe malaria cases [38, 40]. In one study in South Africa, HIV positive patients with severe malaria were significantly more likely to have lactic acidosis than HIV negative patients [17]. Positive HIV status is a known risk factor for complicated malaria, increasing incidence and severity, as well as reducing the likelihood of successful treatment [17–20]. Although blood lactate concentration was strongly associated with mortality in this study, the effect of HIV on outcome was not significant when controlling for lactate levels.

Despite the advantages of readily available and effective medication, these benefits cannot be fully realised if children are delayed in reaching medical care. Extended coma duration prior to admission at hospital is an indicator of this delay to presentation in CM patients. Determinants of delay in seeking treatment for uncomplicated malaria have been studied in Ethiopia, Nigeria, Equatorial Guinea, and Tanzania [41–45], and the primary contributors are household duties and dynamics, socioeconomic status, and transportation problems. In Tanzania, diagnosis and treatment of uncomplicated malaria within 24 h of the onset of symptoms reduced progression to severe malaria and was associated with decreased mortality [41]. These previous studies considered uncomplicated malaria, whereas this is the first to investigate the effect of coma duration prior to admission on clinical outcomes in CM specifically.

Coma duration prior to hospitalization surfaced as the most significant variable associated with sequelae in this analysis. A prolonged delay prior to appropriate hospital care could be due to multiple reasons. More than half of the patients whose comas lasted for ≥ 24 h prior to admission to hospital were linked to an institutional cause associated with the health care system and referral routes (Fig. 2, Table 3).

When considering the effect of delay to presentation on clinical outcome, it was originally hypothesised that a longer delay would lead to higher rates of both mortality and sequelae. On finding that there was a distinct contrast in the way the two outcomes were influenced, the differences between them became a point of interest for the study and two additional hypotheses were considered:

1. Clinical outcome evolves on a spectrum, from infection to death, with full recovery and variable states of survival with neurosequelae in between. This hypothesis assumes that all outcomes would be influenced by the same driving factors.
2. There are different causal pathways leading to full recovery, sequelae, and death, each with their own distinct drivers. This hypothesis assumes that the outcomes have independent pathways.

The results from the multinomial regression analysis suggested that the variables of interest influenced death and neurological sequelae differently, supporting the second hypothesis.

This study has the advantage of using data from one of the longest standing studies of the pathogenesis of CM, spanning 20 years and including over 1600 well-characterized cases from a single study site. Nevertheless, there were some limitations. Over the course of the study, both the standard of living and health care system in Malawi changed. Increased availability of transport could have made it easier for patients to reach hospital faster, for instance. Increased numbers of staff and more district health care centres may have led to more prompt treatment prior to arriving at the referral hospital. These are potential confounders. In addition, the determination of sequelae was made at discharge only, thus failing to capture both sequelae that developed over time, and the resolution of any sequelae seen at discharge. Extensive follow up data are being collected on a subset of these patients and further analysis will be informative. Data on time to presentation was collected via questionnaire completed by the parent or guardian in conjunction with research staff. Reporter bias might be inherent, with parents or guardians tending to minimize the amount of time that they delayed prior to taking action. In addition, the individual providing the history at the hospital may not be the same as the one present during the initial phases of the disease: for example, aunts and grandmothers often accompany the patient to the hospital when the mother is occupied at home with housework and care for the patient’s siblings. In addition, the parents or guardians may have an inaccurate perception of ‘coma’. These all lead to the measure of ‘coma duration prior to admission’ being a variable to be interpreted with care. In addition, there are possibly a subset of children that die quickly prior to reaching QECH. It is currently impossible to document these cases given the diagnostic and reporting capabilities in the more rural referral areas. This could lead to a selection bias in the cases being considered at the referral hospital. Each of these weaknesses would decrease the effect on outcome, however the presence of a striking differential effect of coma duration on sequelae and mortality emphasizes that there is still value in considering this variable. Finally, only a small number of comprehensive patient histories were available to determine reasons for delay to presentation, with fifty-seven of these having a coma duration of 24 h or longer.

## Conclusion

This study sheds light on how a delay to presentation at hospital is significantly associated with the clinical outcome of children with CM. It highlights a novel distinction between the pathogenic processes leading to death and sequelae that warrants further investigation. Although the treatment and understanding of CM has improved, there are still barriers to full recoveries. It is critical that children are given medical attention in a timely manner. Beyond parents and guardians taking action to present to health care quickly, it is the responsibility of the country, the health care system, health care workers and institutions to facilitate this rapid presentation. Efforts in the education of caregivers as well as careful public health measures could target these delays and in turn, lead to a decrease in the burden of sequelae in CM survivors.

## Supplementary Information

**Additional file 1: Table S1.** Analysis with multiply imputed datasets: results of the pooled Brant test.

## Data Availability

The datasets used and analysed in this study are available from the first author or corresponding author on reasonable request.

## Abbreviations

CM: Cerebral malaria
HIV: Human immunodeficiency virus
ACT: Artemisinin-based combination therapy
BMP: Blantyre Malaria Project
PRW: Paediatric Research Ward
QECH: Queen Elizabeth Central Hospital
WHO: World Health Organization
IQR: Interquartile range
OR: Odds ratio
CI: Confidence interval
MRI: Magnetic resonance imaging
