# Supplementary material for: Delayed presentation to hospital care is associated with sequelae but not mortality in children with cerebral malaria in Malawi
Source: Malar J. 2022 Feb 22;21:60. doi: 10.1186/s12936-022-04080-2 (PMC8864854; doi:10.1186/s12936-022-04080-2)
Supplement: Supplementary file 1 — Additional file 1: Table S1. Analysis with multiply imputed datasets: results of the pooled Brant test. [file 12936_2022_4080_MOESM1_ESM.docx]

## **Additional file 1**

Table S1: Analysis with multiply imputed datasets: results of the pooled Brant test.

| Test for | Chi-Square Statistic | Degrees of Freedom | P-value |
| --- | --- | --- | --- |
| Omnibus | 22.15 | 6 | 0.001 |
| Age (months) | 5.16 | 1 | 0.023 |
| Coma duration (hours) | 5.82 | 1 | 0.016 |
| HIV status | 0.90 | 1 | 0.342 |
| Prior treatment: other | 0.02 | 1 | 0.902 |
| Prior treatment: artesunate | 0.88 | 1 | 0.349 |
| Lactate at admission (mmol/L) | 7.61 | 1 | 0.006 |
